# Photo‐Driven Ion Transport for a Photodetector Based on an Asymmetric Carbon Nitride Nanotube Membrane

**DOI:** 10.1002/anie.201907833

**Published:** 2019-08-02

**Authors:** Kai Xiao, Bin Tu, Lu Chen, Tobias Heil, Liping Wen, Lei Jiang, Markus Antonietti

**Affiliations:** ^1^ Max Planck Institute of Colloids and Interfaces Department of Colloid Chemistry 14476 Potsdam Germany; ^2^ Laboratory of Theoretical and Computational Nanoscience CAS Key Laboratory for Biomedical Effects of Nanomaterials and Nanosafety CAS Center for Excellence in Nanoscience National Center for Nanoscience and Technology Beijing 100190 P. R. China; ^3^ Key Laboratory of Bio-inspired Smart Interfacial Science and Technology of Ministry of Education School of Chemistry Beihang University 100191 Beijing P. R. China; ^4^ Key Laboratory of Bio-inspired Materials and Interfacial Science Technical Institute of Physics and Chemistry Chinese Academy of Sciences Beijing 100190 P. R. China

**Keywords:** carbon nitride, ion transport, nanoionics, photodetectors, porous membranes

## Abstract

Conventional photosensing devices work mainly by electron processing and transport, while visual systems in intelligence work by integrative ion/electron signals. To realize smarter photodetectors, some photoionic device or the combination of ionic and electronic devices are necessary. Now, an ion‐transport‐based self‐powered photodetector is presented based on an asymmetric carbon nitride nanotube membrane, which can realize fast, selective, and stable light detection while being self‐powered. Local charges are continuously generated at the irradiated side of the membrane, and none (fewer) at the non‐irradiated side. The resulting surface charge gradient in carbon nitride nanotube will drive ion transport in the cavity, thus realizing the function of ionic photodetector. With advantages of low cost and easy fabrication process, the concept of ionic photodetectors based on carbon nitride anticipates wide applications for semiconductor biointerfaces.

## Graphical abstract

**A self‐powered photodetector** based on ion transport is reported that exhibits high performance in transforming a light signal to ionic current, and meets the requirements of the conventional photodetectors: high spectrum selectivity, high signal‐to‐noise ratio, high sensitivity, fast response speed, and high stability.

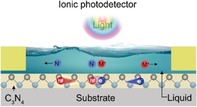

With the enormous potential of commercial and industrial applications and a huge investment in scientific research,^1^ conventional electron‐transport‐based photodetectors, fabricated from vacuum tubes,^2^ organic conducting polymers^3^ or crystalline inorganic semiconductors,1b, 4 have made tremendous progress and are parts of our daily life. Up to now, state‐of‐the‐art electron‐transport‐based semiconductor photodetectors can realize ultrafast, ultrasensitive detection of light in the ultraviolet, visible, infrared‐ and terahertz frequency ranges.^5^ In many aspects, these photodetectors even have better performance than the visual system of living systems.^6^ However, next generation of photodetector system that has the requirements to contact with living matter directly will require the introduction of new features such as bidirectional interfacing and integrated ion/electron signals. From this point of view, ion‐transport‐based photodetectors should be developed to facilitate the development of smart human‐computer interaction,^7^ because biological systems speak a different language: they are mainly dependent on the combination of electronic and ionic signals to detect and interpret information from external stimuli; that is, ion transport and gradients are the indispensable part to collect and transport external signals.^8^, ^9^

In vivo, ions and ion transport regulate the biological processes at the single‐cell scale, enable the propagation of electronic signals, and maintain a suitable balance between the fluids of the extracellular and intracellular environments, which is extremely important for several processes including nerve impulses, hydration, muscle function, and the regulation of pH level.^10^ In vitro, ions transport is also addressed in topics as the solar flow battery,^11^ concentration gradient energy conversion,^12^ and water desalination.^13^ For that, different solid‐state nanopores, nanochannels, and porous membranes have been developed.^14^ High‐performance ionic diodes,14b ionic transistors,^15^ and ion pumps^16^ have been realized in fundamental research. In the views of applied research, diverse ion sensors,^17^ energy conversion and storage systems,^18^ and water desalination systems^19^ are in development to meet the demand of a smart and green society in which ion transport based photodetector or ionic photodetector should be one crucial part of this great aim.

Herein, we proposed a self‐powered ionic photodetector. Conventional electron transport based photodetectors (Figure 1 a) work by the separation of electrons and holes in polymeric carbon nitride activated by incident photons,^20^ while ionic photodetector works by the ions (cation and anion) separation in electrolyte induced by an asymmetric surface charge distribution on a semiconductor surface (Figure 1 b). Indeed, light is first converted into separated charges located along a gradient, which then however induces a flux of mobile ions for charge compensation. The liquid solution character can guarantee reproducible and stable contacts, good biocompatibility, and structural plasticity. In this work, construction of the present ionic photodetector is based on an asymmetric carbon nitride nanotube membrane (ACNNM), which is homogeneously negatively charged in its initial state,^12^ but changes to an asymmetric surface charge distribution when light is shined on it (Figure 1 c). Then, the mobile ions in the electrolyte will move to balance the asymmetric surface charge, which can be understood as a light‐induced ionic current. The ionic photodetector exhibits high performance in transforming the light signal to the ionic current signal, and meet the requirements of the conventional photodetectors: high spectrum selectivity, high signal‐to‐noise ratio, high sensitivity, fast response speed, and high stability.^21^ Furthermore, the ACNNM‐based ionic photodetector can work without an external power source, a remarkable simplification considering potential applications in biological media.

**Figure 1:**
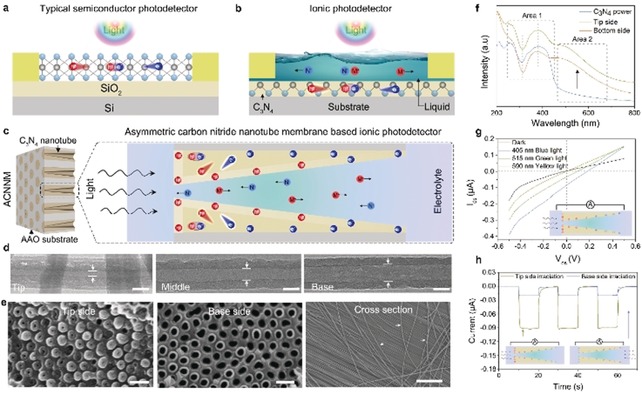
Comparing electronic and ionic photodetectors. a) Diagram of the typical electron‐transport‐based photodetector. b) An ion‐transport‐based photodetector. c) The asymmetric carbon nitride nanotube membrane used in this work and the mechanism of light‐induced ion transport. d) TEM images of ACNNM at tip, middle, and base sides. Scale bar: 50 nm. e) Cross‐sections of ACNNM at tip and base sides (scale bar: 200 nm) and the released carbon nitride nanotube (scale bar: 5 μm). f) UV/Vis absorption spectroscopy of carbon nitride powder and from tip and base sides of ACNNM. g) Typical current–voltage curves of an ACNNM ionic photodetector before and after irradiation with different light. h) Photocurrent responses of the ionic photodetector with tip side irradiation and base side irradiation by 50 mW cm^−2^ blue light.

The asymmetric carbon nitride nanotube membranes were fabricated by the vapor deposition polymerization (VDP) method (Supporting Information, Figure S1).^16^ TEM sections of the tip, middle, and bottom part clearly illustrate the gradually changing diameter with tip‐side average inner diameter 15–20 nm and base‐side average inner diameter 70–80 nm (Figure 1 d). SEM images also showed the top view of the tip side and base side, as well as the long tube (Figure 1 e). The patterns of X‐ray diffraction (XRD) from tip and base parts are consistent with each other (Supporting Information, Figure S2) and confirm carbon nitride molecular structure (Supporting Information, Figure S3).^22^ As show in Figure 1 f, the UV/Vis absorption spectra of ACNNM from tip side and base side are consistent with each other and typical carbon nitride in area 1 (200–450 nm), but have a stronger absorption in area 2 (450–700 nm), which can be attributed to the effect of surface defects.^23^ The enhanced light absorption provides additional possibilities for its application in high performance photodetector.

The ion‐transport properties across ACNNM were measured in a home‐made H‐type cell (Supporting Information, Figure S4), which was filled with 0.01 m KCl solution as a medium. The current–voltage (*I*–*V*) curve in dark showed that our ACNNM has a week rectification ratio about 2 (Figure 1 g dark line; Supporting Information, Figure S5), which can be ascribed to the asymmetric structure and negative charged wall surface.^24^ After unilateral light irradiation in tip side, both the ionic currents on negative and positive potential increased, and the *I*–*V* curve deviates the origin, exhibiting a short‐circuit current and an open circuit voltage; and the signal depends on the incident light energy (405 nm blue light; 515 nm green light; 590 nm yellow light). The observation of a current without a bias potential lays the foundation for its application as a self‐powered ionic photodetector. Furthermore, base‐side irradiation and tip‐side irradiation showed obviously different phenomenon: the ionic current increment from base side irradiation is almost 4 times as large as that from tip side irradiation, which can be explained by two aspects. One reason is that the tip side has a larger irradiated area, resulting in a stronger absorption and enhanced ionic current. Another reason is that base side diameter is much larger than the Debye screening length (about 4 nm for 0.01 m electrolyte), in which way the photoelectric effect will be diluted.^25^

Based on these carefully designed ACNNM, we examined the figures of merit of the ionic photodetector, following the 6S principles: high spectrum selectivity, high signal‐to‐noise ratio, high sensitivity, fast response speed, high stability and self‐power property. Figure 2 a presents the spectrum selectivity of ACNNM‐based ionic photodetectors. The time‐dependent photocurrents were measured at −0.5 V bias potential with same light power density (50 mW cm^−2^), but different wavelengths (blue light: 405 nm; green light: 515 nm; yellow light: 590 nm). It can be seen that the ionic photodetector device exhibits the strongest responsivity to high energy blue light, while a weaker responsivity is found for low energy yellow light. The obvious different responsivity to different light energy follows well the behavior of the absorbance of the semiconducting ACNNM (Figure 1 f).

**Figure 2:**
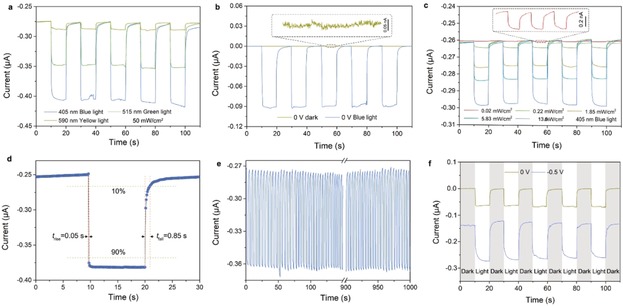
a) Photocurrent responses of the ionic photodetector at different light illuminations (blue, green, yellow, power density: 50 mW cm^−2^) show high spectrum selectivity at −0.5 V bias. b) Photocurrents of the ionic photodetector measured in dark and under light illumination (50 mW cm^−2^ blue light at 0 V bias) show a high signal‐to‐noise ratio. c) Time‐dependent photocurrents of ACNNM based ionic photodetector as a function of incident light power show high sensitivity (Blue light at −0.5 V bias). d) The measured photocurrent responses indicate a rise time of less than 0.05 s and a fall time of less than 0.85 s, indicating a fast sensing speed. e) Photoresponse of the ionic photodetector for 330 cycles (1000 s) illustrate the high stability. f) Photocurrent response of the ionic photodetector at different bias (yellow: 0 V; blue: −0.5 V) means the ionic photodetector can still work without an external power source (0 V).

The ACNNM‐based ionic photodetector also exhibits a high signal‐to‐noise ratio. Figure 2 b shows the dark current and photo current with 50 mW cm^−2^ blue light irradiation at 0 V bias. In general, the dark current at 0 V bias is less than 0.02 nA, defined as the off state (which is maybe much smaller, but is limited by the sensitivity of the used instrument), while a photodetector under illumination is defined as the on state, which is about 0.1 μA. The current ratio between on and off states (on/off ratio) reveals the sensitivity of the photodetector to certain irradiation.^21^ For the ACNNM‐based ionic photodetector, the on/off ratio can reach 5000, which is high compared with conventional photodetectors.^26^

Figure 2 c depicts a representative set of the time‐dependent currents under light illumination with different incident power at −0.5 V bias potential. Following light illumination, the current gain shows a clear power dependence (Supporting Information, Figure S6). The photocurrent is calculated by *I*_pc_=*I*_light_−*I*_dark_, where *I*_light_ and *I*_dark_ are the ionic current under light illumination and dark, respectively. The responsivity is given by *R*=*I*_pc_/*P*, where *P* is the incident light power density. Within the incident power range of 0.04 to 15 mW cm^−2^, the photodetector shows a linear response with photocurrent from 1 nA to 0.1 μA, while the responsivity decreases from 30 μA W^−1^ to 3 μA W^−1^. We have to state that the responsivity of our ionic photodetector has no advantage compared with other photodetectors,^27^ but has much room to improve by increasing ion transport rate, for example by increasing electrolyte temperature or going to quantum tunneling fluid.^28^

Fast response to optical signals, which is here coupled to of charge (electrons or ions) transport and collection, is critical for optoelectronic devices.^29^ Figure 2 d shows the temporal photocurrent response of the ACNNM‐based ionic photodetector. The measured switching times for the rise (current increasing from 10 % to 90 % of the peak value) and fall (current decreasing from 90 % to 10 % of the peak value) of the photocurrent are 0.05 s and 0.85 s, respectively, indicating a rather fast response speed. It is an obvious disadvantage that due to the involved liquid transport of ions, the response time of ionic photodetector must be much slower than an electronic photodetector, which can obtain response times of several nanoseconds.5b, 30 However, it is fast enough to meet most of the photoelectric devices and can be further improved by increasing ions transport speed. The fact that the decrease is significant longer than the increase is due to the relaxation of the photoinduced surface charges within the semiconductor, which is obviously rather long‐lived. Furthermore, the ionic photodetector is a four‐step signal conversion process: light signal is converted into surface localized photocharges which created an ionic current to be finally read out in an electronic signal. We predict that the ionic photodetector should also be faster and has a higher responsivity when it is been used in some localized ion readout device, just like synapses and neurons.^31^

Stability is one of the most important characteristics of optoelectronic devices. Figure 2 e shows the response of photocurrent to optical pulses at a time interval of about 3 s. We found that the dynamic photoresponse of the ionic photodetector was stable and reproducible at least on a 1000 s time scale. For about 330 cycles, the photocurrent quickly increases as soon as the light is turned on and then drops to the original value when the light is turned off, and this process experiences no obvious changes. Furthermore, some devices were retested after several weeks and displayed the same photodetector effect (Supporting Information, Figure S7).

The ability to run without any power supply is also highly appealing, especially in wet and biological environments.^32^ The time‐dependent current curves of the ACNNM device under 50 mW cm^−2^ blue‐light illumination (Figure 2 f) showed similar photoelectric response performance at any bias potential of −0.5 V and without bias. This suggests that the ionic photodetector can work without external power (0 V) as well as with power supply (−0.5 V). Meanwhile, we also noticed that the photoinduced current gain under 0 V is smaller than that under −0.5 V. For the −0.5 V bias, the current gain is about 0.14 μA, while the current gains is about 0.075 μA under 0 V bias. This is due to the fact that the light generates charge pairs in the semiconductor nanotubes, while the applied potential bias when applied in the correct direction improves charge separation.^33^ In other words: the semiconductor nanotube acts as a photovoltaic cell driving its own ability to sense light.

Importantly, the working conditions of our ionic photodetector are rather broad. It can work at different electrolyte concentrations, different electrolyte pH, and different electrolytes. In a range of electrolyte concentration from 10^−6^ m to 0.1 m (Figure 3 a; Supporting Information, Figure S8), the photocurrent gain is about constant. Photocurrent gains at different pH from 3 to 11 are also very similar (Figure 3 b). Different electrolytes including monovalent, divalent, and trivalent cations were also used at different light conditions and show no obvious difference to each other (Figure 3 c). However, all the current gains of ionic photodetector under various electrolyte conditions show obvious difference to different light, following well the behavior of the absorbance of ACNNM (Figure 1 f).

**Figure 3:**
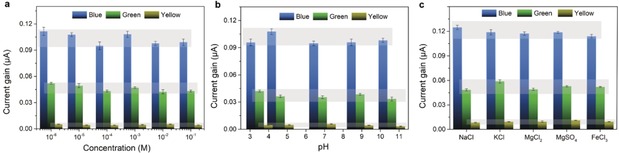
Current gain of ionic photodetector under a) various electrolyte concentrations, b) various electrolyte pH, and c) various electrolyte species when illuminated by blue, green, and yellow lights, respectively.

Surface charge redistribution of ACNNM before and after light irradiation is thought to be the key of the ionic photodetector. Before illumination, the ACNNM is negatively charged because of the electron rich −NH imidic groups,^12^ which are generated in C_3_N_4_ polymerization and condensation process (Supporting Information, Figures S9, S10). After unilateral illumination, local charges are continuously generated at the irradiated side of the membrane, and none (fewer) at the non‐irradiated side, which results in a surface charge gradient (Supporting Information, Figures S11, S12). Therefore, the illuminated side is positively charged, and the whole conical nanochannel is inhomogeneously charged, which will then induce ion transport in solution to balance the inhomogeneous surface charge distribution.^34^ The inhomogeneous surface charge distribution can be expressed by the transmembrane potential. Figure 4 shows the potential difference across the ACNNM. Before illumination, the potentials in base and tip sides have no obvious difference, while after illumination potential difference jumps to about 0.5 V and decreases to zero again when turning down the light irradiation. These phenomenon can also be confirmed by PNP (Poisson and Nernst–Planck) calculations (Supporting Information, Figures S13, S14).

**Figure 4:**
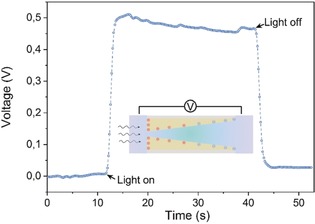
Time‐dependent voltage curve across ACNNM showing the obvious transmembrane potential after illumination at 0 V bias.

In summary, an ion‐transport‐based photodetector was realized based on asymmetric carbon nitride nanotube membrane. Photoinduced separation of electrons and holes results in the asymmetric surface charge within the cavity surface of the nanotube, which is the base of the working principle of the ionic photodetector. The present ionic photodetector has the merit of high spectrum selectivity, high signal‐to‐noise ratio, high sensitivity, fast response speed, and high stability. Meanwhile, it can work without external power supply and in any electrolytes. We speculate that such a carbon nitride nanotube‐based photovoltaic device without any external power source can be used in bioelectronic applications, such as an artificial retina. In comparison with other electron‐transport‐based photodetectors, the ACNNM‐based ionic photodetector is cheap, easy to fabricate, and extendable.

## Conflict of interest

The authors declare no conflict of interest.

## Supporting information

As a service to our authors and readers, this journal provides supporting information supplied by the authors. Such materials are peer reviewed and may be re‐organized for online delivery, but are not copy‐edited or typeset. Technical support issues arising from supporting information (other than missing files) should be addressed to the authors.

Supplementary
