# Supplementary material for: Photo‐Driven Ion Transport for a Photodetector Based on an Asymmetric Carbon Nitride Nanotube Membrane
Source: Angew Chem Int Ed Engl. 2019 Aug 2;58(36):12574–9. doi: 10.1002/anie.201907833 (PMC6790565; doi:10.1002/anie.201907833)
Supplement: Supplementary file 1 — Supplementary [file ANIE-58-12574-s001.pdf]

Angewandte Chemie
Eine Zeitschrift der Gesellschaft Deutscher Chemiker

Supporting Information

Photo-Driven Ion Transport for a Photodetector Based on an Asymmetric Carbon Nitride Nanotube Membrane

Kai Xiao, Bin Tu, Lu Chen, Tobias Heil, Liping Wen, Lei Jiang, and Markus Antonietti*

anie_201907833_sm_miscellaneous_information.pdf

1. Experimental section:

General: Unless otherwise noted, all of the commercial reagents were used as received. Melamine (purity >98.0%) was purchased from Sigma-Aldrich. Target 60-μm-thick AAO membrane with pore width 84 ± 15 nm were purchased from Heifei Puyuan Nano, China. Glass test tube for vapor-deposition polymerization (VDP) were purchased from Merck Millipore. Blue, green, and yellow LED light were used for light irradiation, respectively. In this work, unless otherwise noted, all the light illumination were provided by blue LED light. *I-V* curves and constant voltage ionic current were adjusted to zero current at zero voltage to remove small offsets experienced between runs. All measurements were carried out at room temperature. The main transmembrane potential used in this work was stepped at 0.05 V/step for 1 s/step (0.05 V/s) from -0.5 to +0.5 V, with its period of 21 s.

Fabrication of ACNNM. The carbon nitride nanotube membrane was fabricated by a vapor-deposition polymerization (VDP) method described before.[1] Firstly, the commercial AAO membrane (Diameter: 5 mm) were cleaned by ethanol and deionized water, then dried by nitrogen. Then, the cleaned AAO were put into the bottom of the glass test tube in a certain direction (Supporting Figure1) to get an asymmetric structure. The samples were placed in the oven to heat to 773 K with a heating rate of 10 K/min, and then keep for 4h to insure the sufficient polymerization. After the temperature naturally cooled dawn to room temperature, the AAO membrane tuned from transparent white to brown, and yellowish carbon nitride power at the bottom of the test tube can be obtained. To get a pure carbon nitride nanotube for TEM or SEM, the carbon nitride nanotube membrane was immersed in 1M acid for chemical etching (72 h), then cleaned by deionized water and dried in 60 °C oven.

Ionic photodetector properties. The ions transport and ionic photodetector properties were studied by measuring the current-voltage curves and constant voltage ionic current through the ACNNM with and without light illumination. ACNNM membrane was mounted between two chambers of a home-made H cell, which are full of electrolytes. The cell has a transparent glass window for light irradiation. Ag/AgCl electrodes were used to collect the current and voltage signals. Open circuit voltage (photovoltage) was measured by electrochemical work station (Gamry interface 1000). Ionic current (photocurrent) was measured by a Keithley 6430 picoammeter (Keithley Instruments, Cleveland, OH). To the light density dependent measurements, the light density can be controlled by controlling the distance between light source and ACNNM. And light power intensity was measure by portable light intensity meter.

Calculation. The trans-nanotube potential was systematically analyzed by a theoretical model based on Poisson and Nernst-Planck (PNP) equations with proper boundary conditions,[2]

$$\nabla^2\Phi = -\frac{F}{\varepsilon}\sum z_i c_i$$

$$j_i = D_i \nabla c_i + \frac{z_i F}{RT} D_i c_i \nabla \Phi$$

$$\nabla \cdot j_i = 0$$

Where Φ, c_i, D_i, j_i, z_i are, respectively, the electrical potential, ion concentration, diffusion constant, ionic flux, and charge of species i. ε is the dielectric constant of the electrolyte solution. The diffusion coefficients for cations and anions are 2.0×10^{-9} m^2/s (we use KCl electrolyte for simplicity). The boundary condition for potential Φ on the nanotube wall is,

$$\vec{n} \cdot \nabla \Phi = -\frac{\sigma}{\varepsilon}$$

where σ is the surface charge density. And the surface charge density is various along with our experiment condition. The ion flux has the zero normal components at boundaries,

$$\vec{n} \cdot j = 0$$

The geometric parameters are in Fig. S13. The stationary solver was generally used. But when it fails, the parametric solver was applied. For all the calculations, the accuracy is set to be less than 10^{-6}.

2. Characterizations

The released CNNs was transferred to a quartz glass substrate and analyzed. The scanning electron microscope (SEM) JSM-7500F (JEOL) at an accelerating voltage of 3 kV was used to get the top view and cross section of the CNNs. X-ray diffraction (XRD) patterns were recorded with a Bruker D8 Advance instrument with Cu Kα radiation. Shimadzu UV 2600 was used to reveal the optical absorbance spectra of CNNs and powders. Surface photovoltaic spectroscopy was measured by a surface photovoltaic spectrometer (CEL-TPV1000). The membrane Zeta Potential was measured with SurPASS 3, Anton Paar.

3. Supporting datas

Figure S1. The fabrication of asymmetric carbon nitride nanotube membrane.

Figure S2. XRD of ACNNM from both tip and base sides.

Figure S3. FT-IR spectra of ACNNM.

Figure S4. Home-made testing H-cell.

Figure S5. Asymmetric current-voltage curve of ACNNM.

Figure S6. Photocurrent and responsivity as a function of incident light power.

Figure S7. Stable photocurrent responses of the ionic photodetector.

Figure S8. Ionic currents at different concentration electrolytes with different light irradiation.

Figure S9. Zeta potential of ACNNM.

Figure S10. The streaming potential of the carbon nitride nanotube membrane.

Figure S11. The schematic of light transmission with and without 450 nm blue light irradiation.

Figure S12. The transmitted light spectrum.

Figure S13. Calculation nanotube model (not in scale).

Figure S14. Mechanism of ionic photodetectors.

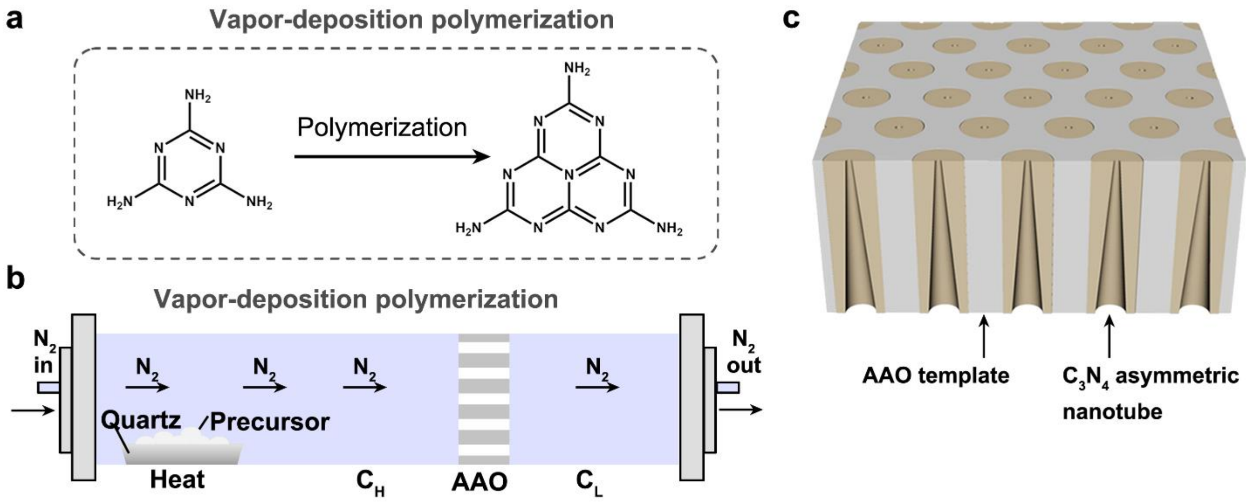

Figure S1. The fabrication of asymmetric carbon nitride nanotube membrane. a, Vapor-deposition polymerization chemical process. **b,** In fabrication, the AAO membrane was used as a template, which can separate the reaction chamber to two different areas. The area with precursor will has a high carbon nitride concentration (C_H) while another area has a low concentration (C_L). In the polymerization and condensation process, asymmetric carbon nitride was generated. **c,** Schematic form of C_3N_4 asymmetric nanotube membrane in AAO template.

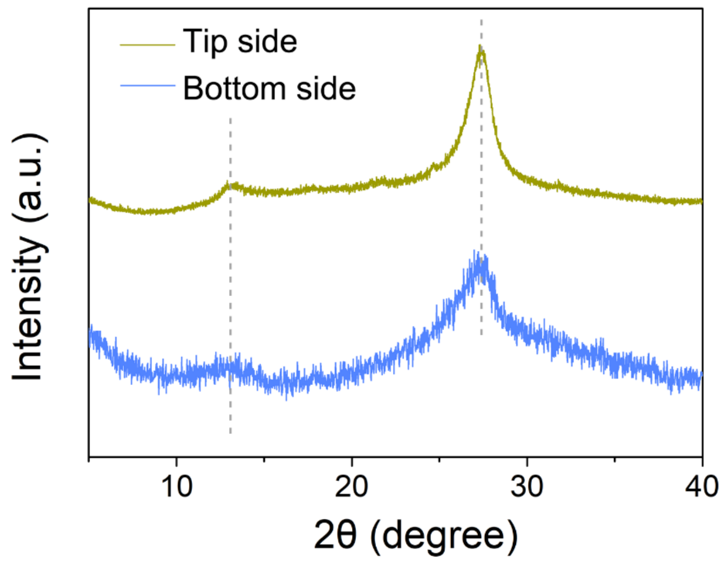

Figure S2. XRD of ACNNM from both tip and base sides.

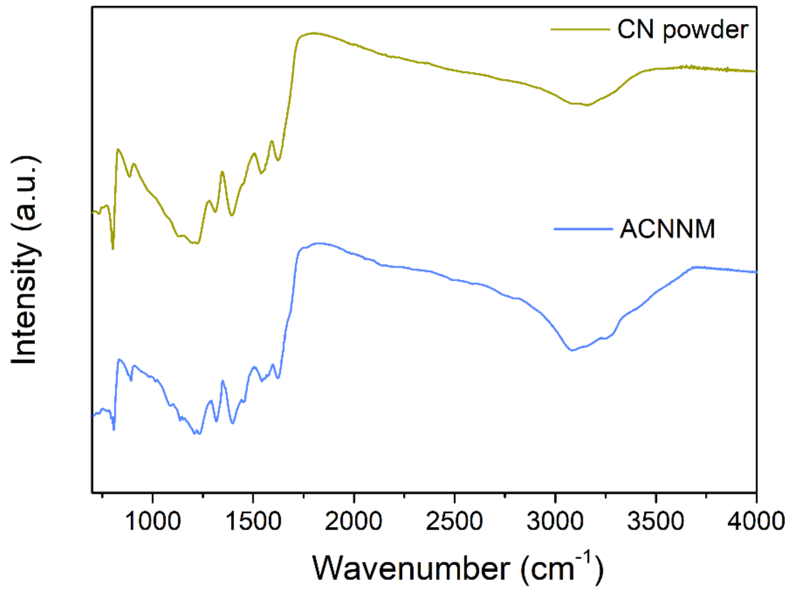

Figure S3. FT-IR spectra of ACNNM. The typical stretching modes of CN heterocycles were found at 1200 to 1600 cm^{-1}. Moreover, the characteristic breathing mode of the triazine units was found at approximately 800 cm^{-1}, thus indicating the formation of C_3N_4 nanotube membrane.

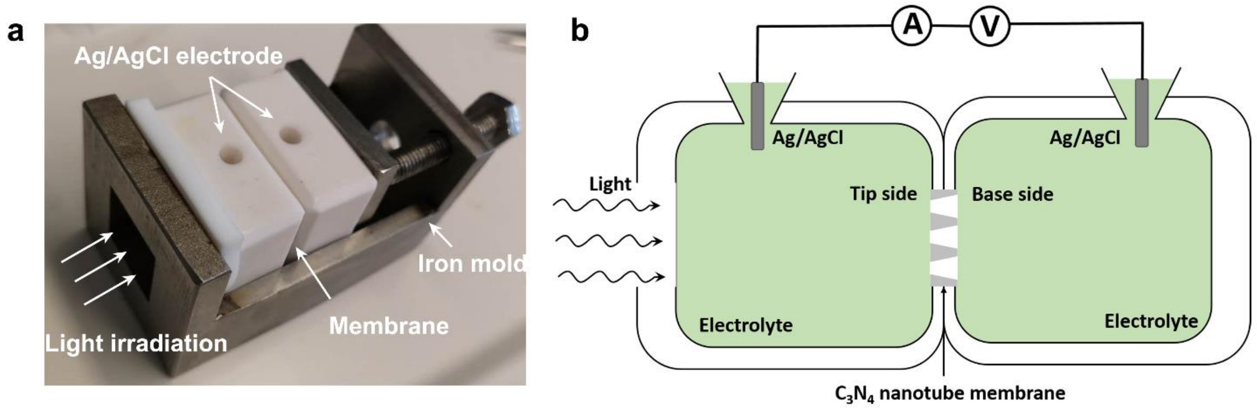

Figure S4. a, Home-made testing H-cell. **b,** Schematic form of the test device.

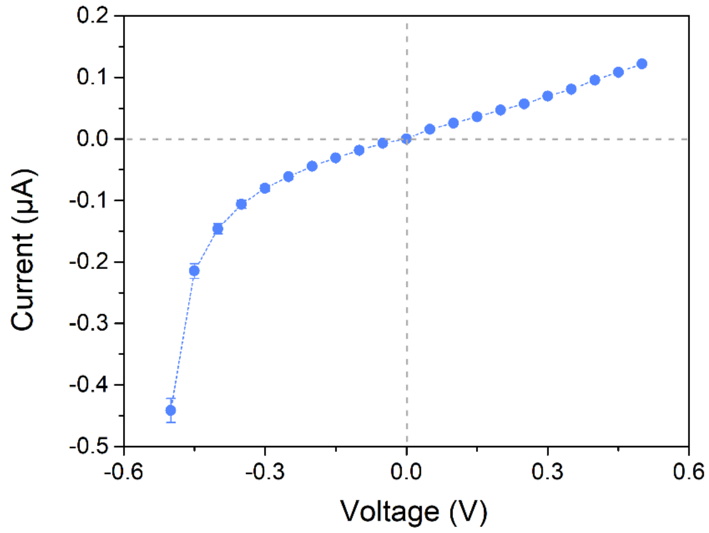

Figure S5. Asymmetric current-voltage curve of ACNNM, resulted from the asymmetric structure.

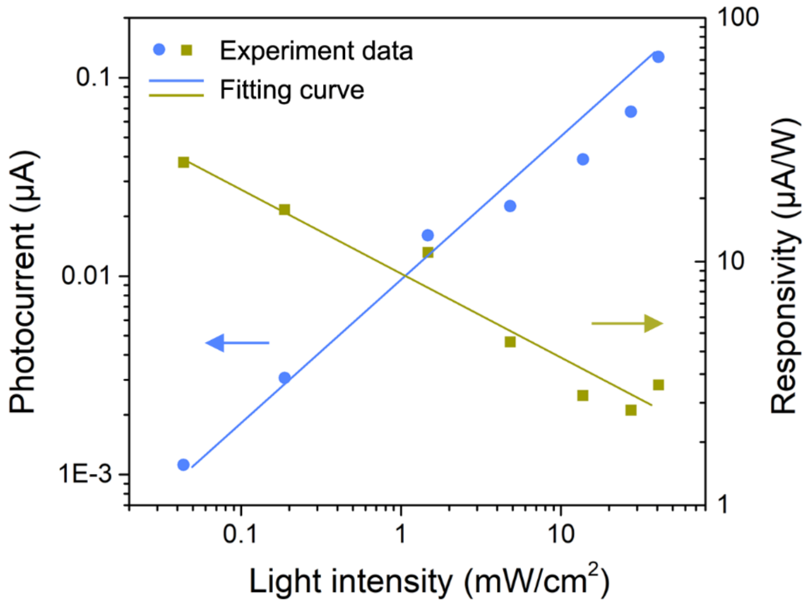

Figure S6. Photocurrent and responsivity as a function of incident light power.

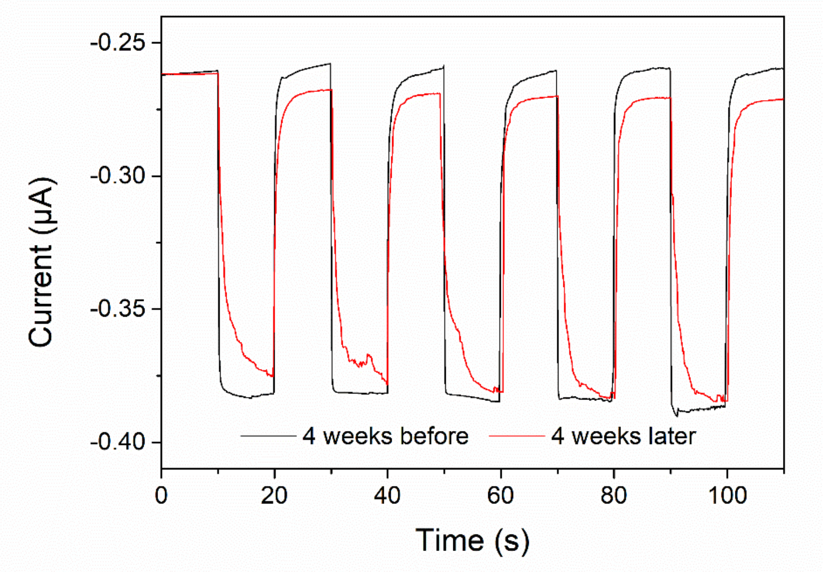

Figure S7. Photocurrent responses of the ionic photodetector before and after four weeks, which show no obvious difference with each other.

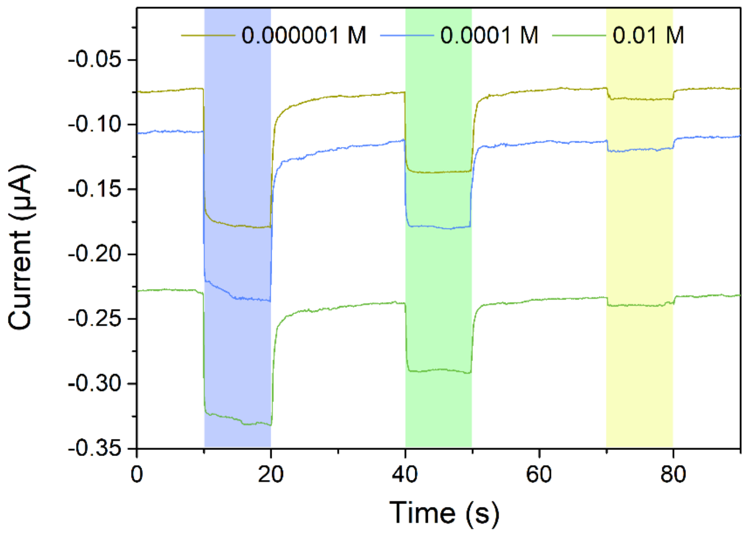

Figure S8. Ionic currents under constant voltage at different concentration electrolytes with different light irradiation.

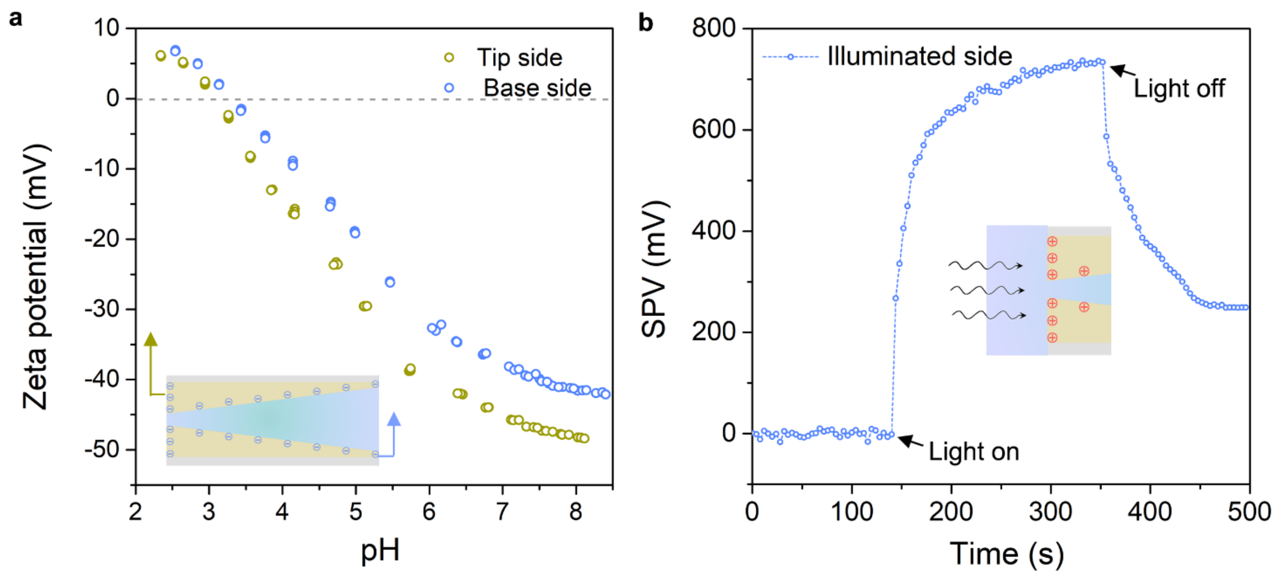

Figure S9. a, Zeta potential of ACNNM in both sides shows its negative charged surface before illumination. **b,** Surface photo voltage (SPV) of ACNNM in illuminated side indicates its positive charged surface.

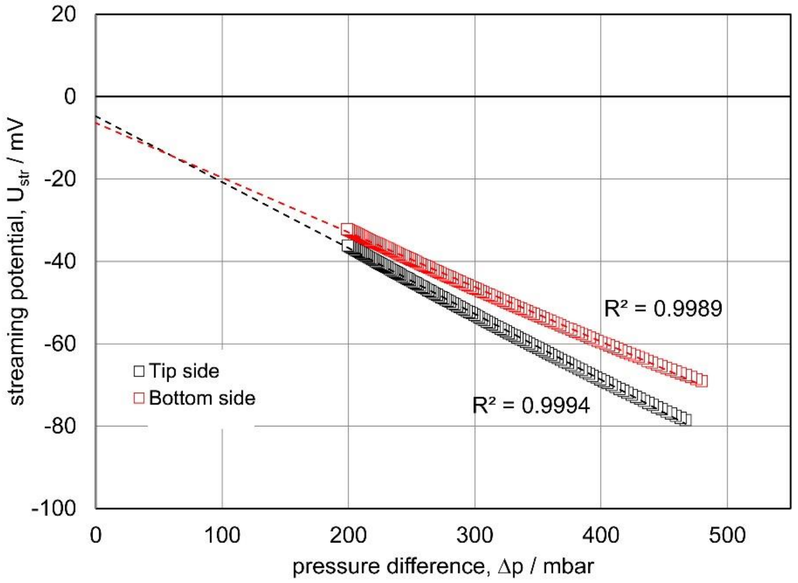

Figure S10. The streaming potential of the carbon nitride nanotube membrane as a function of pressure difference. Both the potential from tip side and bottom side can confirm the original carbon nitride nanotube membrane is negative charged.

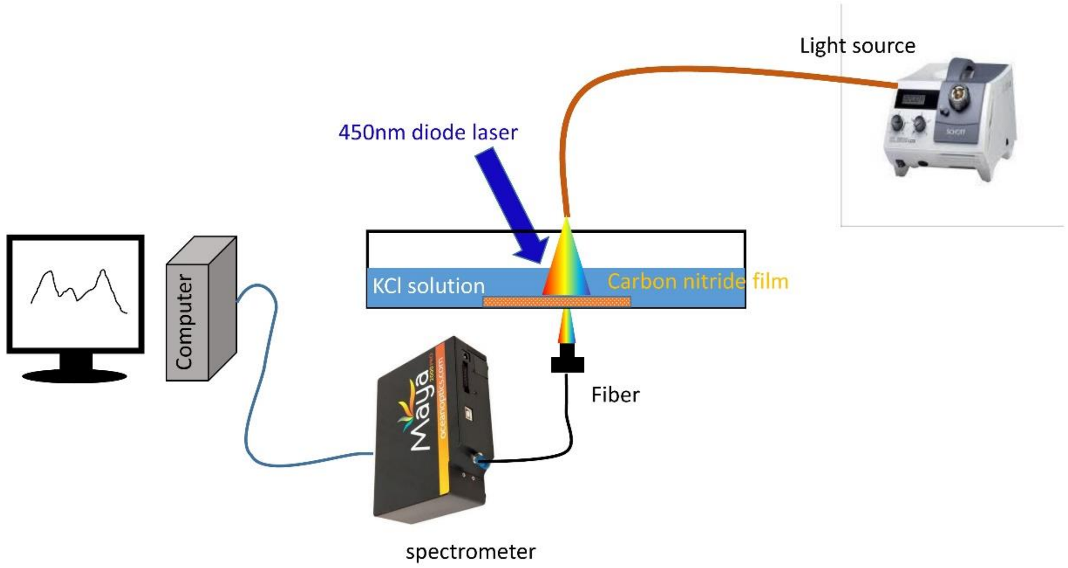

Figure S11. The schematic of light transmission with and without 450 nm blue light irradiation. The work principle is as follows: the light source (KL2500 LCD) provided an all-optical wavelength light to the carbon nitride nanotube membrane in KCL solution (0.01 M), in which part of the light was absorbed by membrane while part transmitted the membrane to be detect by spectrometer (Maya ocean optics). Then, we studied the transmitted light signal with and without the 450 nm diode laser (10 mW/cm^2).

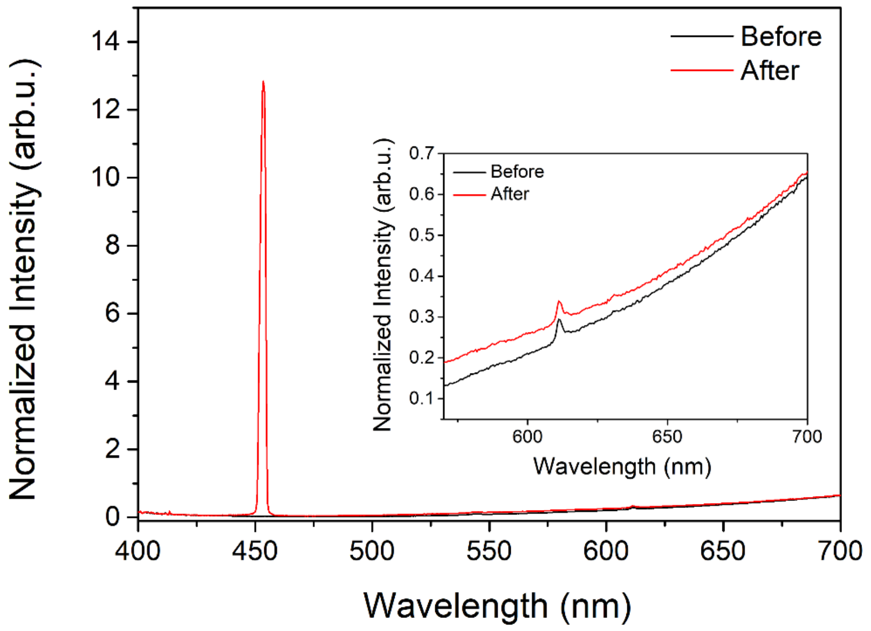

Figure S12. The transmitted light spectrum. There are two obvious differences before and after the 450 nm diode laser. One is the 450 nm peak, which should be from the laser. Another one is the transmitted light intensity after the 450 nm diode laser has an obvious enhanced. This can be understood as widening of the band gap upon laser irradiation. The 450nm beam kicks the electrons out from the valence band to the conduction band, which causes further absorption of the UV-Vis photons more difficult (UV vis absorption).

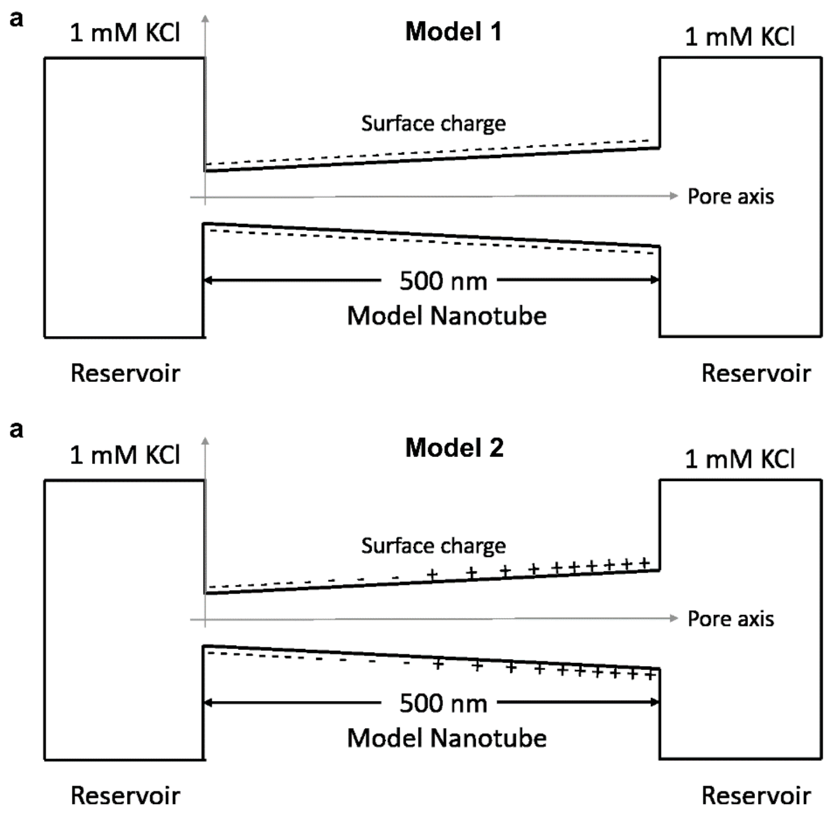

Figure S13. Calculation nanotube model (not in scale). a, model 1 with symmetric negative charge surface; **b,** model 2 with asymmetric charge surface. The theoretical simulation is based on the coupled two-dimensional Poisson–Nernst–Planck equations within the commercial finite-element package COMSOL 5.1 script environment.

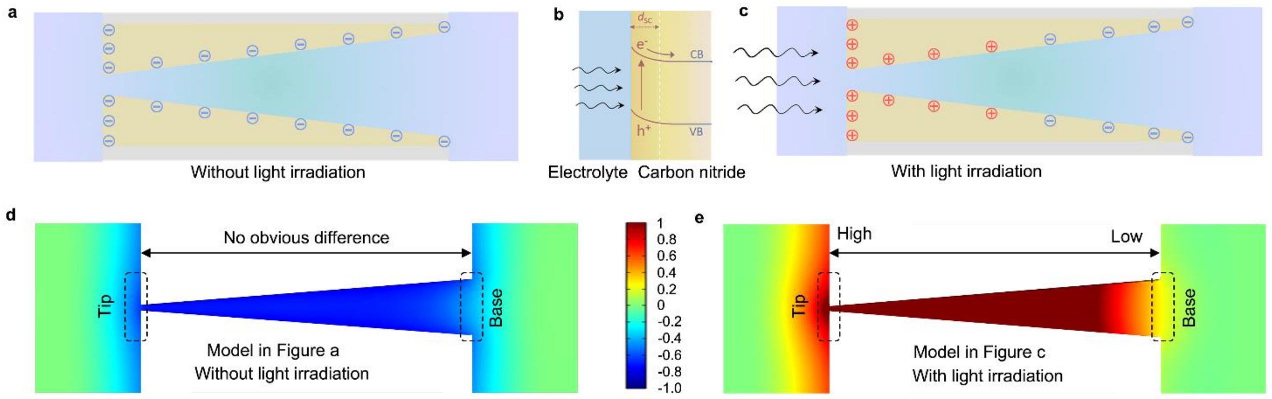

Figure S14. Mechanism of ionic photodetectors. a, Schematic of negative charged surface of ACNNM without illumination. **b,** Photo-induced separation of electrons and holes of carbon nitride, and the resulted band bending at the surface of the semiconductor phase. **c,** Schematic of asymmetric surface charge distribution in ACNNM after light irradiation. **d, e,** Calculated potential distribution across ACNNM without (d) and with (e) light irradiation at 0 V bias.

4. Reference

1. Xiao, K.; Chen, L.; Chen, R.; Heil, T.; Lemus, S. D. C.; Fan, F.; Wen, L.; Jiang, L.; Antonietti, M., *Nat. Commun.* **2019,** *10*, 74.
2. White, H. S.; Bund, A., *Langmuir* **2008,** *24*, 2212-2218.
